# The effect of hydroxyethyl starches and crystalloid fluids on mortality and need for dialysis in vascular surgery patients

**DOI:** 10.1186/2197-425X-3-S1-A744

**Published:** 2015-10-01

**Authors:** R Green, S Hicks, M Butler

**Affiliations:** Nova Scotia Truama Program, Dalhousie University, Halifax, Canada; Department of Critical Care, Dalhousie University, Halifax, Canada; Department of Emergency Medicine, Dalhousie University, Halifax, Canada

## Introduction

Previous research has indicated that fluid choice is an important component of the resuscitation of the critically-ill patient. Synthetic hydroxyethyl starches (HES) may remain within the intravascular space compared to crystalloid solutions, yet they have also been associated with acute kidney injury. Data comparing HES and crystalloid solutions in anesthesia is incomplete. The goal of this study was to evaluate the effect of utilizing HES on adverse patient outcomes in vascular surgery patients at a Canadian academic hospital.

## Objectives

To compare the association of the administration of HES solutions with crystalloid solutions on in-hospital mortality and the requirement for hemodialysis in patients that underwent a vascular surgery procedure.

## Methods

This was an unblinded, retrospective review of 796 patients that underwent a vascular surgery procedure between 2007 and 2009. The patients were divided into those that received HES intraoperatively (n = 213) and those that received crystalloid fluids (n = 583). The data were abstracted from an electronic intraoperative information system, a hospital central patient registry, and the patient record. Inclusion criteria were all adult (≥ 16 years) patients that were intubated for a vascular surgery procedure.

To assess for an association between in-hospital mortality, hemodialysis requirement, ventilator requirement and inotrope requirement we utilized a logistic regression model that adjusted for gender, age, urgency of procedure and American Society of Anesthesiologists (ASA) score.

## Results

Receiving HES solutions was associated with a greater post-operative hemodialysis requirement (OR = 19.53, CI: 1.54, 247.0), inotrope requirement (OR = 2.74, CI: 1.48, 5.08), ventilator requirement (OR = 8.50, CI: 5.40, 13.40), and intensive care unit (ICU) admission (OR = 9.81, CI: 6.51, 14.77). Patients that received HES solutions were more likely to be admitted to both an in-patient ward and an ICU. Receiving HES solutions was not associated with in-hospital mortality (OR = 1.50, CI: 0.77, 2.91).

## Conclusions

In our study of vascular surgery patients of varying acuity, administration of HES was associated with poor patient outcomes compared to crystalloid solutions. More study is required to fully elucidate the effects of HES on in-hospital mortality and hemodialysis requirements of this patient population.

